# Validation of the Bangla version of Beck Depression Inventory‐II

**DOI:** 10.1002/brb3.1563

**Published:** 2020-02-11

**Authors:** Sheikh Md. Abu Hena Mostafa Alim, Md. Nazir Ahmed, Mohammad S. I. Mullick, Nafia Farzana Chowdhury, Farzana Akhter, Md. Shamsul Alam

**Affiliations:** ^1^ Department of Psychiatry Rajshahi Medical College Rajshahi Bangladesh; ^2^ Department of Psychology Chittagong College Chittagong Bangladesh; ^3^ Department of Psychiatry Bangabandhu Sheikh Mujib Medical University Dhaka Bangladesh; ^4^ Department of Psychology University of Rajshahi Rajshahi Bangladesh; ^5^ Department of English Dhaka City College Dhaka Bangladesh

**Keywords:** Bangladesh, cognitive, depression, diagnosis, Diagnostic and Statistical Manual of Mental Disorders, factor analysis, translation

## Abstract

**Background:**

Beck Depression Inventory (BDI‐II) is a widely used valid instrument to assess the severity of depression in clinical and normal settings. To meet the necessity of a standard scale for measuring depression among above 265 million Bangla speaking population around the world, this scale was translated and validated.

**Methods:**

Two translations of BDI‐II into Bangla were prepared, and then, two back translations were done by medical and language experts in parallel. Thereafter, sentence revision followed by pretest on 20 respondents was done to finalize the Bangla version of BDI‐II (BDI‐II BV). Afterward, a cross‐sectional, comparative, and descriptive study was conducted to validate the scale by purposive sampling technique consisting of 111 persons (both clinical and normal) in three tertiary‐level hospitals in Bangladesh. Everyone was given to fill up BDI‐II BV at first. Then, they were given to fill up BDI‐II BV (*n* = 49), Bangla version of Depression Anxiety Stress Scales 21‐item (DASS21‐BV, *n* = 47) and BDI‐II (*n* = 25) 3–7 days later. The diagnosis of depressive disorder was made according to DSM‐5. Correlation study and factor analysis were completed.

**Results:**

The mean age was 28.83(±8.70) years. The male–female ratio was 1:0.82. Correlation of scores for BDI‐II BV with the DASS21‐BV depression subscale was .920; BDI‐II BV with BDI‐II was .985 (Cronbach's *α* .993; *t* test not significant) and BDI‐II BV applied first and the second time was .960 (Cronbach's *α* .979; *z* test not significant). The interitem correlation for all the items was found highly significant (*p* < .01). Patients having depressive disorder or episodes had significantly higher BDI‐II BV scores than normal (*M* + *SD* 30.18 + 10.127 than 8.34 + 5.910; *p* < .001). Partial confirmatory factor analysis demonstrated two‐factor loading comprising Cognitive and Somatic‐affective symptoms.

**Conclusions:**

Through the translation and validation process, a validated Bangla version of BDI‐II was produced to measure depression and its severity among the Bengali population.

## Graphical abstract

BDI‐II Bangla version is highly correlated with original English, and Interitem correlation for all the items was found highly significant. It retained two‐factor loading comprising Cognitive and Somatic‐affective symptoms. Through the translation and validation process, a validated Bangla version of BDI‐II was produced to measure depression and its severity among Bengali population.

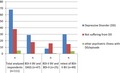

## 1 INTRODUCTION

Depressive disorder and comorbid symptoms of depression with other physical and mental illnesses are very common psychiatric morbidity around the Globe and so also in Bangladesh. Bengali (Bangla) is counted as the seventh most spoken native language in the world by population (Central Intelligence Agency [CIA], 2019). The majority of Bengalis among 265 million Bangla speakers worldwide are living in Bangladesh (Eberhard, Simons, & Fennig, 2019). The number of patients suffering from depression is gradually increasing day by day. Therefore, we need a reliable tool in Bangla to measure the severity of depression to combat this most common mental health problem. As specific laboratory investigation is lacking to confirm the diagnosis and severity assessment for depression (Soron, 2017), validated Bangla version of a rating scale for depression assessment like Beck Depression Inventory‐II (BDI‐II) could meet this requirement.

According to WHO (2017), the prevalence of depressive disorders in Bangladesh was 4.1%. An increasing trend was shown in the National Mental health survey where depressive disorder was reported as 6.7% (Directorate General of Health Services [DGHS], 2019). As stated by the Institute for Health Metrics and Evaluation (IHME) (2017), depressive disorders in Bangladesh ranked third in the status of years lived with disability (YLDs) both in 2007 and in 2017 with a 21.8% increase in number at the last time. People of Bangladesh mostly express their depression or sadness through somatic symptoms along with psychological symptoms (Chowdhury, 1979; Salim, 2010). So a scale that can measure somatic symptoms of depression, as well as cognitive and affective symptoms, should be used to measure the severity of depression among the Bengalis.

Beck Depression Inventory‐II is one of the most widely used gold standard valid instruments to assess the severity of depression in clinical and normal settings developed by Aaron T. Beck, Robert A. Steer, and Gregory K. Brown (Cusin, Yang, Yeung, & Fava, 2010). This can be administered to individuals aged from 13 to 80 years. Administration time is about 5–10 min (Beck, Steer, & Brown, 1996). This second edition of the Beck Depression Inventory features new items that brings it in line with criteria for diagnosing depressive disorders of the Diagnostic and Statistical Manual of Mental Disorders—fourth edition (DSM‐IV) and specifically constructed to measure the severity of self‐reported depression in adolescents and adults (Beck, Guth, Steer, & Ball, 1997). It is used as an indicator of the presence and degree of depressive symptoms, not as an instrument for specifying a clinical diagnosis. The 21‐item BDI‐II covers both somatic and psychological (cognitive and affective) symptoms measuring items. Initial studies of the BDI‐II have shown it to possess a high degree of internal consistency. Some studies, using outpatients, found the mean coefficient alpha to be .92 for the BDI‐II (Beck et al., 1996; Steer, Ball, Ranieri, & Beck, 1997; Steer, Clark, Beck, & Ranieri, 1999). As there is no crucial change has been made in the diagnostic criteria of Depression & Depressive Disorders in DSM 5, BDI‐II can be well fitted with this edition. It is found that the BDI‐II has been translated into several languages, including Arabic, Chinese, Dutch, Finnish, French, German, Icelandic, Italian, Japanese, Persian, Spanish, Swedish, Turkish, and Xhosa (Smarr & Keefer, 2011). It has also been translated and validated into the Malay language in Malaysia where there is similarity in religious and cultural ambiance with Bangladesh (Mahmud, Awang, Herman, & Mohamed, 2004). Again, one of our SARK countries Sri Lanka has also validated this scale in Sinhalese (Rodrigo, Kuruppuarachchi & Pathmeswaran, 2015).

In recent years, researchers of Bangladesh and West Bengal of India have given scientific endeavors to translate and validate few internationally accepted scales to measure depression into Bangla, for example, Bangla version of the Depression Anxiety Stress Scales 21‐item (DASS21‐BV) (Alim et al., 2014), Bangla Montgomery Asberg Depression Rating Scale (MADRSB) (Soron, 2017), and Bengali adaptation of Edinburgh Postnatal Depression Scale (EPDS‐B) (Gausia et al., 2007; Maity, Saha, Sanyal, & Biswas, 2015). As DASS21‐BV is a multidimensional scale that was validated only among medical students, its use among the patient is uncertain. On the other hand, the clinician‐rated Montgomery Asberg Depression Rating Scale (MADRS) was developed in the late 1970s to measure the severity of depression in clinical settings (Montgomery & Asberg, 1979). Because this scale was never updated or modified, it does not target reverse neurovegetative symptoms (Cusin et al., 2010). Moreover, Self‐rating scales, such as the BDI, offer some advantages over clinician‐rated scales, as they may take less time, do not require trained personnel, and their administration and scoring process appear more standardized (Biggs, Wylie, & Ziegler, 1978).

As written by Soron (2017); researchers also tried to translate BDI‐II and validate the Bangla version; but the attempted Bangla version of the BDI lacked sound methodology as the question raised for its permission from the author, copyright and ethical approval related pitfalls. However, no journal article was found regarding the validation of the Bangla version of BDI‐II to date.

As there are a few scales for measuring depression among the Bangla speaking population especially in Bangladesh, research regarding this issue would be helpful to reduce physical in addition to mental symptoms and improve quality of life as well as an overall economic burden to patients having depressive disorder or comorbid depression. This study was carried out for adaptation and validation of the Bangla version of BDI‐II for measuring the severity of depression among the Bangladeshi population.

## 2 MATERIALS AND METHODS

The research was conducted in two phases, translation phase and validation phase. As prior permission from the copyright holder NCS Pearson, INC was obtained after paying licensure fees for translation and validation, no additional approval was taken from the author according to the patent rules. Ethical clearance was obtained from the Ethical Committee of Mental Hospital, Pabna; permission was taken from appropriate authorities of particular hospitals. Copies of permission letter from NCS Pearson, Inc. and ethical clearance were sent to the editor of this journal.

### 2.1 Translation phase

This segment was carried out from March to October 2015. The translation process was according to guidelines stipulated in the US Census Bureau Guideline (Pan & Puente, 2005) and standards of the American Psychological Association (2014).

#### 2.1.1 Forward translation and backward translation

Two forward translations into Bangla were done by a psychologist having sound knowledge regarding depression along with its sociocultural variation in Bangladesh and another by a professor of English expert in translation. Then, first translation was given to a psychiatrist and second to another professor of English for backward translations who were blind to the original English version of BDI‐II. All persons involved in the translation process were Bangladeshi having sound knowledge in both Bangla and English language.

#### 2.1.2 Expert review

Sentence revision was done by experts involved in the translation process in a panel discussion after the reconcilement of the forward and backward translations. Then, a multidisciplinary expert committee comprised of psychiatrists, psychologists, a psychosocial worker, language experts, and all the translators reviewed original version and translated materials of BDI‐II in meetings. At the end of these processes, a preliminary Bangla version of BDI‐II (BDI‐II PBV) was produced.

#### 2.1.3 Pretest

Beck Depression Inventory‐II PBV was pretested (tryout) among 20 adult literate individuals in Dhaka (both patients and healthy persons) of either sex, that is, 11 males and 9 females, having different sociodemographic characteristics (eight service holders, three housewives, three students, and six others) to identify any flaws in BDI‐II PBV. The respondents were requested in writing whether they could understand the items and comment on each of the items for improvement. Their suggestions were discussed with the experts and necessary modifications were done. At the end of the pretest, a harmonized Bangla version of BDI‐II (BDI‐II HBV) was obtained.

### 2.2 Validation phase

#### 2.2.1 Face and content validity

Beck Depression Inventory‐II HBV was given to a panel of reviewers consisting of two Professors of Psychiatry, a Professor of English and an Assistant Professor of Clinical Psychology at different universities. After reviewing BDI‐II HBV, the BDI‐II Bangla version (BDI‐II BV) was finalized.

#### 2.2.2 Study description

This was a cross‐sectional, comparative, and descriptive study conducted from November 2015 to September 2016. Places of the study were Mental Hospital, Pabna, department of Psychiatry of Bangabandhu Sheikh Mujib Medical University Hospital and Rajshahi Medical College Hospital. Informed consent was taken from the respondents.

#### 2.2.3 Participants

The researchers reviewed the literature extensively but no single agreement regarding sample size determination of a validation study was found. According to Gorsuch (1983), the minimum ratios of participants to items should be 5:1 or 10:1 and it has been cited in different psychological research (Soron, 2017). Although the greater sample size could produce better analysis, due to constraints of funding and time, participants to items ratio were decided to keep about 5:1.

A heterogeneous group of samples consisting of outdoor or admitted patients, their caregivers, hospital staffs of different categories was taken from the above‐mentioned hospitals to validate the BDI‐II BV among both the clinical and nonclinical individuals. The sampling technique was purposive. Bengali speaking and by born Bangladeshi from age 14 to 70 years of both sexes having at least primary level education (fifth‐grade) were included. Disoriented, agitated, mute, stuporous, psychotic and having significant neurocognitive impairment were excluded. Although 126 persons were approached, a total of 115 persons gave consent for the study.

#### 2.2.4 Measures

Both English and Bangla versions of Beck Depression Inventory‐II (BDI‐II and BDI‐II BV) and Bangla version of Depression Anxiety Stress Scales 21‐item (DASS21‐BV) were used in this study (Alim et al., 2014). Diagnosis of depressive disorders and other psychiatric illness were done according to DSM‐5 Criteria (American Psychiatric Association, 2013).

The original English version of BDI‐II consists of 21 items to measure the severity of depression. Each item is a list of four statements arranged in ascending order of severity about a particular symptom of depression which could be rated from 0 (symptom not present) to 3 (symptom strongly present), with resulting summary scores ranging from 0 to 63. The time reference for the response set has 2 weeks. The severity rating guidelines and cutoff score suggested by the authors for total scores of patients diagnosed with major depression are 0–13 minimal; 14–19 mild; 20–28 moderate; and 29–63 severe (Beck et al., 1996). It can be scored manually or by software. The severity rating guidelines of this translated version BDI‐II BV were kept as same as BDI‐II. BDI‐II BV questionnaire already has some demographic questions to collect information regarding age, gender, employment status, education level, and marital status. All participants were requested to fill up that demographic questionnaire.

Original DASS was developed in English by Lovibond and Lovibond (1995) of the University of New South Wales, Australia to measure depression, anxiety, and stress among the respondents. The short‐form version of the Depression Anxiety Stress Scales (DASS21) was created by the same researchers having 21 questionnaires, which can be used instead of the full version of DASS. This valid set of the instrument has seven questions for each subscale of depression, anxiety, and stress (Henry & Crawford, 2005). During the use of the full version of DASS, for depression 0–9 is normal while 10–13, 14–20, 21–27, and 28+ indicate mild, moderate, severe, and extremely severe, respectively. For the short (21‐item) version, multiplication of sum by 2 is needed (Central & Eastern Sydney PHN, 2015; Psychology Foundation of Australia, 2014).The DASS21 was translated and validated into Bangla by Alim et al. (2014), and its Depression subscale was used in this study. The severity rating was retained the same as the original version.

#### 2.2.5 Procedure

Respondents were divided into three groups purposively. All of them were given BDI‐II BV at the start. The first group consisted of 52 persons who were given the Depression subscale of DASS21‐BV at first sitting. The second set comprised of 27 respondents having a good academic background in English was chosen to give BDI‐II original English version 3–7 days apart. Either or the following criteria were applied to be deemed as good in English (GPA 5 in English in 12‐grade final examination or A level, medium of education was English in Graduate or postgraduate level or obtained ≥6 in IELTS). Other than the second group, all the respondents were offered to fill up BDI‐II BV again 3–7 days apart for reliability assessment. The participants were diagnosed by a psychiatrist according to DSM 5 criteria whether they were suffering from depressive disorder or not in the broad sense; depressive episode with other disorders was also assigned.

#### 2.2.6 Statistical analysis

The scoring of all the questionnaires was done manually. Pearson's correlation was measured, and Cronbach's alpha was obtained; *z* test and *t* test were done. Principal component analysis using Oblimin with Kaiser Normalization rotation method and Maximum Likelihood with the above rotation method was used for partial confirmatory factor analysis (PCFA) to extract factors. Statistical Package for Social Science (SPSS) version‐16 and Microsoft Excel (Office 2010) were used to analyze the data and prepare the figures.

## 3 RESULTS

### 3.1 Descriptive analysis

Among the 115 respondents, 111 people completed BDI‐II BV at first setting; 47 people filled up both BDI‐II BV and Depression subscale of DASS, 25 persons completed both the BDI‐II BV and BDI‐II, as well as 49 individuals, took part in test–retest of BDI‐II BV. Hence, analyses were done accordingly. The male–female ratio was 1:0.82. The age range was 14–55 years. The level of education was from grade six to Ph.D., and 54.6% were married. Sociodemographic characteristics are shown in Table 1. Among the total analyzed sample, 65.77% were suffering from depressive disorder or episode (61.26% from depressive disorder, 1.80% bipolar disorder currently depressed, and 2.70% substance‐related disorder with depressive disorder). It was found that 61.7%, 32%, and 61.2% of respondents were suffering from depressive disorder from the first, second, and third groups, respectively. The disease profile is presented in Figure 1.

**Table 1:** Sociodemographic characteristics of the sample

	Total (*n* = 111)** ^,^ #		BDI‐II BV and DASS21‐BV Depression subscale (*n* = 47)* ^,^ #		BDI‐II BV and BDI‐II (*n* = 25)		Test–retest of BDI‐II BV (*n* = 49)	
Age (years)								
Mean ± *SD*	28.83 ± 8.697		31.68 ± 9.146		33.24 ± 8.022		27.02 ± 8.774	
Minimum–Maximum	14–55		17–55		20–55		14–53	
	*n*	%	*n*	%	*n*	%	*n*	%
Sex								
Male	61	55	24	51.1	15	60.0	26	53.1
Female	50	45	23	48.9	10	40.0	23	46.9
Education								
5th to 9th grade	18	16.5	9	19.6			6	12.2
10th to 12th grade	49	45	12	26.1	4	16.0	30	61.2
Graduate and above	42	38.5	25	54.3	21	84.0	13	26.5
Occupation								
Farming	1	0.9					1	2.0
Service	35	32.1	20	43.5	19	76.0	11	22.4
Business	9	8.3	3	6.5			4	8.2
Homemaker	17	15.6	10	21.7	1	4.0	6	12.2
Student	39	35.8	10	21.7	4	16.0	23	46.9
Unemployed	5	4.6	1	2.2	1	4.0	3	6.1
Others	3	2.8	2	4.3			1	2.0
Marital status								
Married	59	54.6	27	61.4	18	72.0	22	44.9
Unmarried	48	44.4	16	36.4	7	28.0	27	55.1
Widow/widower	1	0.9	1	2.3				

* Missing value in education and occupation was 1.

** Missing value in education and occupation was 2.

# Missing value in marital status was 3.

**Figure 1:**
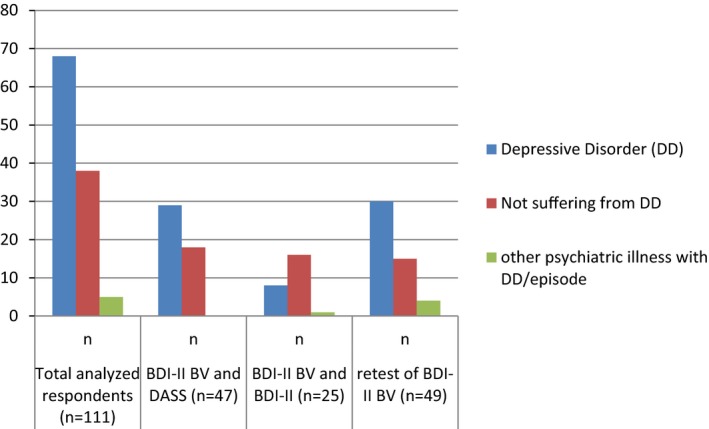
Descriptive for depressive disorder or episode among the sample

### 3.2 Internal consistency

Cronbach's Alpha Based on Standardized items for BDI‐II BV was .926, and intraclass correlation coefficients (ICC) using an absolute agreement definition was .92. The interitem correlation for all the items was highly significant at the .01 level (two‐tailed). These discoveries are portrayed in Tables 2 and 4, respectively.

**Table 2:** Convergent and concurrent validity, reliability, and test of significance in means

	Pearson's correlation	ICC average measures	Cronbach's *α*	*Z* test			*T* test		
				*z*	*p *(*Z*<=*z*) two‐tail	*z* Critical two‐tail	*t*	*df*	*p*
BDI‐II BV first time (*n* = 111)		.920*	.926						
BDI‐II BV and DASS21‐BV Depression subscale (*n* = 47)	.920*	.939*	.959						
BDI‐II BV and BDI‐II (*n* = 25)	.985*	.992*	.993				1.193	24	.244
BDI‐II BV (*n* = 49)test–retest	.960*	.979*	.979	0.2618	.7938	1.9599			
BDI‐II BV among diagnosed case^a^ (*n* = 73) and nonsufferer (*n* = 38)							12.235	109	<.001

a Suffering from depressive disorder or episode.

* 1% level of significance (*p* < .01, two‐tailed).

### 3.3 Convergent validity

Pearson's correlation for BDI‐II BV and DASS21‐BV depression subscale was .920 (Findings are depicted in Table 2). A comparison of severity shows an almost similar result for BDI‐II BV with DASS21‐BV Depression subscale. As BDI‐II BV does not have an extremely severe option in severity rating, that option of the DASS21‐BV Depression subscale is merged with the severe rating. These findings are given in Table 3.

**Table 3:** Cross tabulation of BDI‐II BV with DASS21‐BV depression subscale, BDI‐II and DSM 5 criteria for depressive disorder or episode

	BDI‐II BV severity				BDI‐II BV for screening		Total
	0–13 minimal	14–19 mild	20–28 moderate	29–63 severe	0–13 nondepressed	14–63 depressed	
DASS21‐BV depression subscale severity							
Normal (0–9)	13	3	1	0			17
Mild (10–13)	1	1	1	0			3
Moderate (14–20)	1	3	0	0			4
Severe (21–27+)	0	0	2	21			23
Total	15	7	4	21			47
BDI‐II severity							
0–13 minimal	14	1	0	0			15
14–19 mild	0	3	0	0			3
20–28 moderate	0	0	4	0			4
29–63 severe	0	0	0	3			3
Total	14	4	4	3	33	70	25
DSM 5 criteria							
No depressive disorder or episode					30	8	38
Depressive disorder or episode					3	70	73
Total					33	78	111

### 3.4 Criterion (concurrent) validity

As BDI‐II was designed to measure the severity of depression, it was expected that the BDI‐II BV score would be higher in patients with depressive disorder or episode. Cross tabulation among DSM 5 diagnosis and BDI‐II BV severity measures were done assuming minimal (0–13) as nondepressed and above scores of all severity (14–63) as depressed. Among the 73 diagnosed cases of depressive disorder or episode by DSM 5, 95.89% scored above 13 and among the 38 nondepressive disorder persons, 78.95% were within minimal (nondepressed) severity of BDI‐II BV. Those who were suffering from depressive disorder or episode had significantly higher BDI‐II BV scores (*M* ± *SD* 30.18 ± 10.127) than those who were not suffering (*M* ± *SD* 8.34 ± 5.910; *p* < .001).

Again, the correlation between the BDI‐II BV score and the BDI‐II score was .985; Cronbach's alpha (based on standardized Items) was .993. Here, *t* test showed no significant difference in means (Table 2). While comparing the severity, it was found that 100% of the sample with minimal, moderate, and severe severity in BDI‐II BV matched with BDI‐II, whereas 75.0% matched with mild. A comparison can be seen in Table 3.

### 3.5 Reliability

Correlation for BDI‐II BV applied 1st and 2nd time was .960; Cronbach's alpha was .979. *Z* test shows no significant difference in means in this group. Pearson's correlation and ICC of all the groups were significant (*p* < .001). The outcomes can be seen in Table 2. Test–retest of BDI‐II BV shows the highest correlation for the item “Loss of interest in sex” and lowest for the item “Past failure.” Again, test‐retest of BDI‐II BV applied at 1st and BDI‐II 2nd time demonstrates the highest correlation for the item “Loss of interest in sex” but lowest for the item “Tiredness or fatigue.” Reliability measurements for individual items are presented in Table 4.

**Table 4:** Interitem reliability statistics

BDI‐II items	BDI‐II BV applied 1st and 2nd time (*n* = 49)			BDI‐II BV applied at 1st and BDI‐II applied at 2nd time (*n* = 25)		
	Interitem correlation	*p*	Cronbach's *α* a	Interitem correlation	*p*	Cronbach's *α* a
1. Sadness	.787*	.000	.881*	.944*	.000	.971
2. Pessimism	.743*	.000	.853*	.949*	.000	.974
3. Past failure	.533*	.000	.695*	.787*	.000	.881
4. Loss of pleasure	.578*	.000	.732*	.686*	.000	.814
5. Guilty feelings	.754*	.000	.859*	.857*	.000	.923
6. Punishment feelings	.866*	.000	.928*	.818*	.000	.900
7. Self‐dislike	.758*	.000	.862*	.884*	.000	.938
8. Self‐criticalness	.673*	.000	.805*	.747*	.000	.855
9. Suicidal thoughts or wishes	.716*	.000	.834*	.858*	.000	.924
10. Crying	.724*	.000	.840*	.804*	.000	.891
11. Agitation	.865*	.000	.928*	.644*	.001	.784
12. Loss of interest	.672*	.000	.804*	.812	.000	.896
13. Indecisiveness	.772*	.000	.872*	.888*	.000	.940
14. Worthlessness	.769*	.000	.870*	.690*	.000	.816
15. Loss of energy	.783*	.000	.878*	.628*	.001	.772
16. Changes in sleeping pattern	.807*	.000	.893*	.697*	.000	.821
17. Irritability	.844*	.000	.915*	.775*	.000	.873
18. Changes in appetite	.808*	.000	.894*	.706*	.000	.828
19. Concentration difficulty	.814*	.000	.898*	.642*	.001	.782
20. Tiredness or fatigue	.738*	.000	.849*	.602*	.001	.751
21. Loss of interest in sex	.917*	.000	.957*	.982*	.000	.991

a Based on standardized items.

* Correlation is significant at .01 level (two‐tailed).

### 3.6 Factorial validity

**Figure 2:**
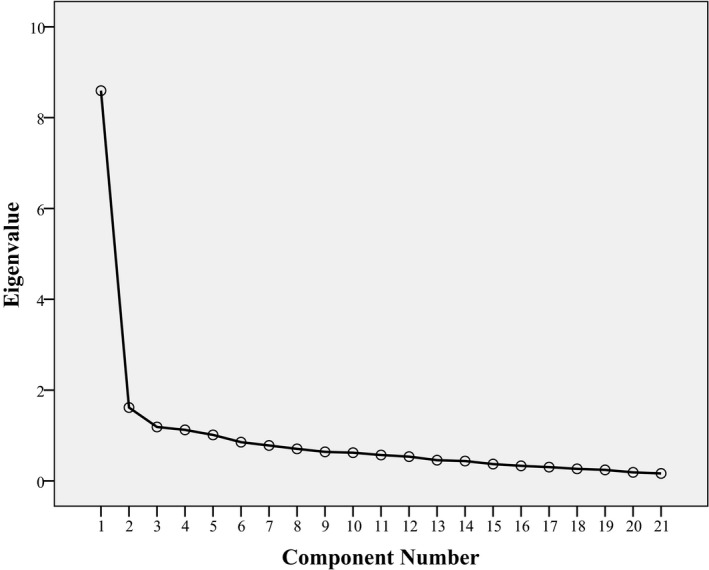
Scree plot showing eigenvalues of factors

The principal component analysis was done on 111 participants using Oblimin with Kaiser Normalization rotation method to determine how many factor solutions might be more appropriate with the present data. Kaiser‐Meyer‐Olkin Measure of Sampling Adequacy was 0.883. Here, five components were extracted with initial eigenvalues 8.583, 1.597, 1.177, 1.163, and 1.024. Here, 1st and 2nd factors cumulatively accounted for 48.477% of the variance in the BDI‐II BV responses (Factor 1 = 40.873; Factor 2 = 7.604%). Applying Cattell's (1966) Scree analysis to the magnitudes of the eigenvalues derived in this analysis suggested that two factors should be extracted (shown in Figure 2). Again Monte Carlo PCA for parallel analysis showed eigenvalues for 1st three components were 1.8626, 1.7091, and 1.5797. So 2 factors were retained and the maximum likelihood with the above rotation method was used for partial confirmatory factor analysis (PCFA). The items that loaded on the first factor were pessimism, past failure, guilty feelings, punishment feelings, self‐dislike, self‐criticalness, suicidal thoughts, or wishes, crying, agitation, loss of interest, indecisiveness, worthlessness, irritability, given that these factors were a composite of mainly cognitive items, it was labeled the Cognitive symptoms of depression. Factor 2 consisted primarily of sadness, loss of pleasure, loss of energy, changes in sleeping pattern, changes in appetite, concentration difficulty, tiredness or fatigue, and loss of interest in sex. As such, this factor appeared to represent Somatic‐affective (emotional) symptoms. Again, these factors were positively correlated with each other {−(−.655)}. Findings are revealed in Tables 5 and 6.

**Table 5:** Interitem correlations of BDI‐II BV (*n* = 111)

Symptoms	Interitem correlations																				
	1	2	3	4	5	6	7	8	9	10	11	12	13	14	15	16	17	18	19	20	21
1. Sadness																					
2. Pessimism	.478																				
3. Past failure	.464	.558																			
4. Loss of pleasure	.587	.476	.426																		
5. Guilty feelings	.324	.407	.381	.426																	
6. Punishment feelings	.352	.302	.358	.381	.541																
7. Self‐dislike	.505	.449	.414	.358	.245	.189															
8. Self‐criticalness	.388	.395	.381	.414	.410	.447	.416														
9. Suicidal thoughts or wishes	.414	.323	.251	.381	.258	.366	.421	.269													
10. Crying	.451	.344	.408	.251	.208	.317	.408	.417	.213												
11. Agitation	.522	.308	.404	.408	.408	.400	.271	.509	.196	.373											
12. Loss of interest	.567	.425	.383	.404	.346	.318	.442	.480	.326	.444	.482										
13. Indecisiveness	.520	.286	.402	.383	.316	.247	.407	.420	.289	.407	.461	.450									
14. Worthlessness	.467	.558	.543	.402	.406	.428	.541	.570	.290	.505	.491	.467	.456								
15. Loss of energy	.469	.435	.429	.543	.335	.311	.450	.306	.307	.260	.414	.348	.339	.510							
16. Changes in sleeping pattern	.560	.315	.314	.429	.102	.160	.216	.255	.269	.329	.379	.380	.377	.286	.351						
17. Irritability	.536	.442	.403	.314	.304	.235	.391	.387	.372	.279	.366	.423	.298	.316	.319	.241					
18. Changes in appetite	.407	.386	.368	.403	.189	.187	.257	.268	.189	.290	.343	.125	.194	.356	.575	.369	.341				
19. Concentration difficulty	.537	.331	.476	.368	.176	.341	.426	.422	.250	.329	.426	.410	.451	.523	.530	.423	.390	.409			
20.Tiredness or fatigue	.629	.487	.365	.476	.206	.320	.394	.303	.317	.330	.437	.342	.358	.458	.666	.449	.384	.552	.585		
21. Loss of interest in sex	.517	.199	.167	.365	.259	.343	.287	.227	.175	.305	.256	.311	.296	.256	.462	.457	.181	.335	.295	.376	
*r_tot_*	.769	.622	.625	.606	.478	.489	.591	.595	.458	.552	.624	.620	.574	.707	.653	.516	.553	.496	.650	.671	.474

Note

*r*_tot_ = corrected item‐total correlation.

**Table 6:** Mean, standard deviation (*SD*) and partial confirmatory factor analysis (PCFA) of BDI‐II BV (*n* = 111)

Symptoms	Mean	*SD*	PCFA (pattern matrix^a^)	
			Factor 1	Factor 2
1. Sadness	1.13	1.019	0.303	−0.566
2. Pessimism	1.05	1.090	0.420	
3. Past failure	1.21	0.926	0.521	
4. Loss of pleasure	1.31	1.060		−0.628
5. Guilty feelings	0.76	0.866	0.667	
6. Punishment feelings	0.99	1.297	0.590	
7. Self‐dislike	1.01	1.040	0.449	
8. Self‐criticalness	1.31	1.212	0.811	
9. Suicidal thoughts or wishes	0.49	0.737	0.307	
10. Crying	1.10	1.198	0.531	
11. Agitation	1.18	0.896	0.522	
12. Loss of interest	1.13	1.105	0.647	
13. Indecisiveness	1.32	1.088	0.515	
14. Worthlessness	1.06	1.130	0.672	
15. Loss of energy	1.07	0.970		−0.726
16. Changes in sleeping pattern	1.09	0.968		−0.543
17. Irritability	0.84	0.920	0.353	
18. Changes in appetite	1.07	1.051		−0.676
19. Concentration difficulty	1.47	0.872		−0.532
20.Tiredness or fatigue	1.26	0.970		−0.907
21. Loss of interest in sex	0.84	1.040		−0.466

a Suppressing absolute value <0.3; rotation converged in 11 iterations.

## 4 DISCUSSION

All the study places were tertiary‐level academic hospitals where inpatient and outpatient psychiatric services along with research facilities were available. Samples were more representative as patients attended in these hospitals from different places and social classes. Exclusion criteria were set to exclude persons from whom data collection could be very difficult. The trained data collectors briefed at the onset regarding the items of the inventory and to some, additional explanations were needed. These indicate that data collectors should have adequate knowledge regarding depression and appropriate training about the application of the instrument while doing future research using BDI‐II BV. While answering, many of the respondents mostly unmarried persons initially did not circle any of the statements of item 21 “Loss of interest in sex.” This might be due to the unexpressive tendency of the people in Bangladeshi culture regarding sex. Furthermore, sexual activities before marriage are socially unaccepted and religiously forbidden. However, this number of respondents was not calculated.

Translations and back translations were done by persons having sound knowledge in both Bangla and English languages. Both linguistic experts and medical experts (psychiatrists and psychologists) were involved in this process. This method was done to ensure the translated version would be grammatically sound and the terms used were correct. At the same time, meanings and contents of original BDI‐II were well preserved. Good translations were reflected by the production of two English back translations, which were almost similar to the original English version. Minor discrepancies were resolved by a panel discussion. Great care was taken to ensure that the translation was culturally sensitive.

Internal consistency of this version was very high which produced similar result with original English where internal consistency on student sample was *α* .93 and among psychiatric sample was *α* .92 (Beck et al., 1996) also in Spanish and Xhosa version where the coefficients were *α* .91 (Wiebe & Penley, 2005) and *α* .93 (Steele & Edwards, 2008)*.* In the study, correlation and ICC between BDI‐II BV with BDI‐II and DASS21‐BV Depression subscale were also very good in addition to BDI‐II BV test–retest reliability. While compared, severity measures were also almost similar to BDI‐II BV and DASS21‐BV Depression subscale. The values of *r*_tot_ ranged from .458 to .769. This compares well with values obtained in the original validation studies reported by Beck et al. (1996) with a large sample of 500 psychiatric outpatients (*r*_tot_ range .39–.70). So it can be said that BDI‐II BV formed a coherent scale and that the items are all selecting a common construct.

Here, two factors, namely Cognitive and Somatic‐affective dimension of self‐reported depressive symptomatology, were loaded which matched with the clinical sample of original (Beck et al., 1996; Steer, Ball, Ranieri, & Beck, 1999) but different in nonclinical student sample by the same researchers (Beck et al., 1996). As more than 60% of the sample in the study was clinically depressed, this corresponds better with the clinical sample of other researchers. However, some minor differences in symptoms loading might be due to different sample characteristics and methods used to analyze the factors.

On score 14 or higher in BDI‐II BV, most of the depressive disorder patients scored positive for depression and the majority of nondepressive disorder persons were excluded. This indicates that BDI‐II BV can be used as a screening tool to detect the depressive disorder. In a Sri Lankan study, it was found that a minimum cutoff score of 16 appeared to provide the best balance between sensitivity and specificity for the Sinhala translation of BDI‐II as a screening instrument (Rodrigo et al., 2015). This difference directs for further research and statistical analysis to determine the cutoff point of BDI‐II BV as a screening tool.

Purposive sampling and lack of divergent validity were the main limitations of the study. Small sample size and study from only centers located in central and northern parts of Bangladesh could hinder the generalization of the result. Clinical interview without any structured questionnaire to diagnosis is another limitation.

## 5 CONCLUSION

Validated BDI‐II BV can be used to measure the severity of depression among Bengali people. Further research is required for its generalization and delineating its psychometric properties among different groups of samples.

## CONFLICT OF INTEREST

About half of the cost of this research was funded by Sun Pharmaceutical (Bangladesh) Limited, which is one of the leading psychotropics such as antidepressant manufacturing pharmaceutical companies in Bangladesh. The rest of the expenditure was borne by the principal author. The remaining authors have no financial disclosures. None of the involved persons neither paid nor received money for this study. The contents are solely the responsibility of the authors and do not necessarily represent the views of the pharmaceutical company.

## Data Availability

Data available on request due to privacy/ethical restrictions.
